# Application of a deep generative model produces novel and diverse functional peptides against microbial resistance

**DOI:** 10.1016/j.csbj.2022.12.029

**Published:** 2022-12-19

**Authors:** Jiashun Mao, Shenghui Guan, Yongqing Chen, Amir Zeb, Qingxiang Sun, Ranlan Lu, Jie Dong, Jianmin Wang, Dongsheng Cao

**Affiliations:** a The Interdisciplinary Graduate Program in Integrative Biotechnology and Translational Medicine, Yonsei University, Incheon 21983, the Republic of Korea; b Department of Biology, School of Life Sciences, Southern University of Science and Technology, Shenzhen 518055, Guangdong, China; c School of Information and Communication Engineering, Hainan University, Haikou 510000, Hainan, China; d Department of Natural and Basic Sciences, University of Turbat, Kech, Turbat, Balochistan 92600, Pakistan; e School of Economics and Management, Southwest Petroleum University, Chengdu 610500, Sichuan, China; f College of Mechanical and Electronic Engineering, Dalian MinZu University, Dalian 116600, Liaoning, China; g Xiangya School of Pharmaceutical Sciences, Central South University, Changsha 410013, Hunan, China

**Keywords:** AMPs, Deep generative model, Antimicrobial resistance, Transformer, LSTM

## Abstract

Antimicrobial resistance could threaten millions of lives in the immediate future. Antimicrobial peptides (AMPs) are an alternative to conventional antibiotics practice against infectious diseases. Despite the potential contribution of AMPs to the antibiotic’s world, their development and optimization have encountered serious challenges. Cutting-edge methods with novel and improved selectivity toward resistant targets must be established to create AMPs-driven treatments. Here, we present AMPTrans-lstm, a deep generative network-based approach for the rational design of AMPs. The AMPTrans-lstm pipeline involves pre-training, transfer learning, and module identification. The AMPTrans-lstm model has two sub-models, namely, (long short-term memory) LSTM sampler and Transformer converter, which can be connected in series to make full use of the stability of LSTM and the novelty of Transformer model. These elements could generate AMPs candidates, which can then be tailored for specific applications. By analyzing the generated sequence and trained AMPs, we prove that AMPTrans-lstm can expand the design space of the trained AMPs and produce reasonable and brand-new AMPs sequences. AMPTrans-lstm can generate functional peptides for antimicrobial resistance with good novelty and diversity, so it is an efficient AMPs design tool.

## Graphical Abstract

**Figure:**
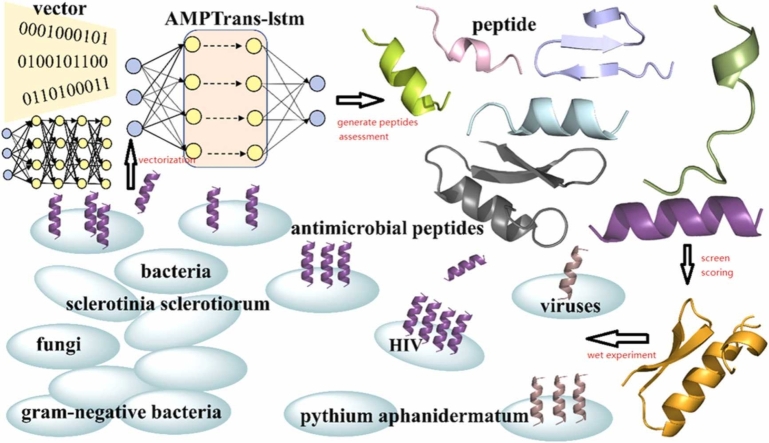

## 1 Introduction

Antimicrobial resistance (AMR) has become a major public health concern mainly either of misuse or overuse of conventional antibiotics in the past several decades, and it threatens the efficient prevention and treatment of microbial pathogens and parasites [1]. If effective solutions are not established, AMR will cause about 10 million human deaths annually by 2050 [2]. The lack of effective antibiotics and the drying antibiotic discovery pipeline have substantiated new approaches to tickle down the vigorously occurring AMR. Among such global platforms to tackle down AMR, substantial efforts have been diverted to develop novel therapeutics to combat AMR including bacteriophage (or phage) therapy, phage-encoded products, immunotherapy and monoclonal antibodies (mAbs) [3]. These novel therapeutic approaches are introduced in preclinical and clinical investigations [4].

Antimicrobial peptide (AMP) is a novel class of alternatives that possess potent activity against a wide range of Gram-negative and positive bacteria with little or no capacity to induce AMR. This has stimulated substantial chemical development of novel peptide-based antibiotics possessing improved therapeutic index. This paper [5] summarizes recent synthetic efforts of AMP, their impact on analog design and their various applications to combat AMR development.

Addressing the dwindling of antibiotics’ efficacy and the rapid emergence of AMR [6], antimicrobial peptides (AMPs) have elicited much attention. Since, AMPs are praised a promising therapeutic strategy due to their rapid and broad-spectrum antimicrobial activities, and low propensity for AMR development. Therefore, the current study develops a deep generative model to design novel AMPs with extended diversity.

As a promising alternative to conventional antibiotics, AMPs are low molecular weight peptides that form a key component of the biological innate immune system. They have the ability to disrupt the cell membranes of target bacteria, interfere with DNA replication, and exhibit broad-spectrum antibmicrobial activities against bacteria, viruses and fungi [7]. They typically consist of up to 100 amino acids, usually short cationic peptides with an alpha-helical secondary structure, and they have amphiphilic surface properties, which are key to their ability to establish antimicrobial activity. AMPs have several advantages over traditional small molecule antibiotics. They have broad spectrum activity for rapid bacterial killing, by disrupting the cell membrane of the target microorganism through hydrophobic or electrostatic interactions and cause cell lysis. AMPs have antibacterial immunomodulatory effects as well as low potential for AMR development [8]. AMPs demonstrate direct broad-spectrum antimicrobial activity and help the immune system combat viruses and bacteria [9]. Compared with small-molecule antibiotics, AMPs are less likely to induce serious bacterial resistance [10]. In addition, AMPs can be used in combination with antibiotics [11] or other drugs for synergistic effects. Addressing the dwindling of antibiotics’ efficacy and the rapid emergence of AMR [6], antimicrobial peptides (AMPs) have elicited much attention.

To date, while more than 15,000 AMPs have been identified and have been successful in managing antibiotic-resistant pathogens, only a small number of AMPs have entered clinical trials [12]. Although AMPs have critical potential in disease management, several of them have limitations associated with their therapeutic applications. The mechanism of AMPs and the design of desirable AMPs for clinical application remains unclear, and why AMPs with considerable structural variations have the same bioactivity is still a mystery. The other limitations of natural AMPs, such as short half-life [13], [14], toxicity to mammalian cells [15], [16], and relatively high production cost relative to conventional antibiotics [17], [18], [19], also hampered their clinical application.

To address the aforementioned issues, several computational approaches have been attempted. Among such approaches, the modeling methods such as molecular dynamics simulations are a powerful tool for antimicrobial study [20], [21], [22], [23]. For example, the mechanistic studies by membrane permeability and molecular dynamics simulation further confirmed the strong membrane interaction and structure of Pardaxin and MSI-78, which contributed to their potent activity [24]. What’s more, the researchers discuss how traditional molecular dynamics simulation works and its role and potential for the development of new antibiotic candidates with an emphasis on antimicrobial peptides[25].

However, manual modification and optimization through expert and molecular dynamics are time consuming, laborious, and challenging [26], [27]. Furthermore, designing AMP candidates that mitigate the aforementioned shortcomings is difficult. Therefore, efficient methods need to be successfully developed for AMP-based therapeutics.

Notably, acquiring and identifying the antimicrobial properties of AMPs through wet experiments are difficult. Although these experimental data are a scarce resource and many AMP databases, such as DRAMP and CAMP [28], [29], have been established, but they are still insufficient.

Deep generative models have exhibited excellent performance in molecular generation and molecular optimization [30], [31]. Christina Wang et al. adopted a long short-term memory (LSTM)-based model to generate novel AMPs [8]. Müller et al. presented a generative LSTM network for peptide design [32], and Tucs et al. proposed PepGAN that achieves balance between covering active peptides and avoiding inactive peptides [33]. Ferrell et al. proposed the AMP-GAN model, a modified conditional generative adversarial network, to design novel AMPs [34]. Meanwhile, Szymczak et al. proposed HydrAMP, a conditional variational autoencoder that learns a lower-dimensional, continuous chemical space of peptides and captures the antimicrobial properties [35]. The main objective of a generative model is to generate novel molecules from chemical spaces obtained from atom-based sequences by learning from the training set. The unique properties of the generated molecules can be further optimized by incorporating conditional constraints. A peptide can be represented in several ways, including chemical fingerprints, simplified molecular input line entry specification, amino acid sequence, and 3D coordinates. As input information, the amino acid sequence obtains the chemical semantics and can be processed by natural language processing methods, such as Transformer and LSTM [36], [37].

In general, these approaches usually have their own merits and demerits. For instance, LSTM is suitable for training on small and medium-sized datasets and has some limitations on the length of molecular sequences. In parallel, despite of its advantages in the stability and validation of molecular generation, LSTM is not efficient at generating innovative molecules.

In contrast, Transformer model is efficient at learning on large datasets and virtually has no limitation on the length of molecular sequences. In addition, Transformer is also capable to generate the innovative molecules. Despite of aforementioned advantages, Transformer has a critical shortcoming, which is the lack of validation of molecules. Furthermore, Transformer is effective at learning long sequences, but it needs a large number of molecules in long sequences, which is often difficult to be satisfied in reality.

In order to take the advantage of the strength and avoid the limitations of both the models, we have designed the current study.

In this study, we have collected data from 12 AMP databases, namely, APD, BACTIBASE, CyBase, DADP, DBAASP, dbAMP, EnzyBase, LAMP, Peptaibol, PhytAMP, YADAMP, and AVPdb [38], [39], [40], [41], [42], [43], [44], [45], [46], [47], [48], [49]. Our model was pre-trained on public databases RCSB PDB [50] and UniProt 2021_04 [51] by learning the chemical semantic relations harbored in the amino acid sequence(s). Then, we have performed transfer learning on our small data set, and the fine-tuned model was trained to produce a biologically appropriate amino acid sequence. The pre-trained model could also generate an improved peptide sequence with a specific function contained in the small data set. After training, QSAR (Quantitative structure–activity relationship) [52], [53] models were used to identify the properties of peptides in a database that is yet to be experimentally validated.

Finally, the newly generated sequences could be identified and classified using sub-structure similarity and trained machine learning models to predict the functions of these new sequences. Thus, the generated novel peptide sequences were assigned candidate antibacterial functions, and some potential lead peptides were recommended for the development of potential antimicrobial peptides as potential therapeutic drugs.

## 2 Methods and models

In this section, we discuss the details of data preprocessing, training data for the model, and the use of AMPTrans-lstm model for AMP design. We show that the proposed AMPTrans-lstm model can be used to generate novel AMP candidates after training and has similar physiochemical properties as the data used for training. Moreover, the desired therapeutic functions of the candidate antimicrobial peptides were preserved by incorporating functional peptide data as specific constraints.

### 2.1 Training data

**Fig. 1:**
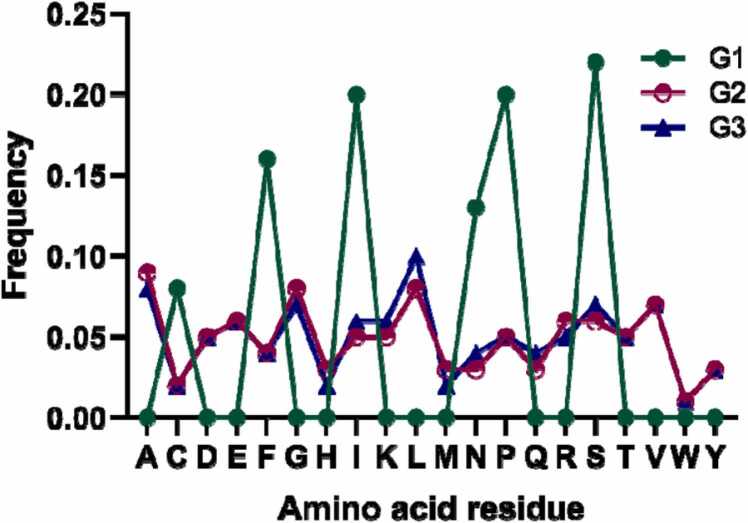
Amino acid distribution in the original trained sequence and sampling from the trained LSTM and Transformer models (G1: generated sequence by Transformer, G2: generated sequence by LSTM, and G3: original sequence).

The pre-training set was designed by combining the databases PDB v2 and UniProt 2021_04 then fine-tuning our collected dataset, as shown in Fig. 1. We extracted sequence information and other information in FASTA format, including microbial targets such as Gram-negative bacteria, Gram-positive bacteria and viruses, activity measures (mainly MIC50 in μg/mL) and mechanism targets (cytoplasmic proteins, cell membranes and cell replication), when they were available. Data preprocessing was performed to analyze the training sequences and filtered for sequences containing non-FASTA symbols, such as lowercase characters and tail modifications, etc., and for sequences with more than 2000 amino acid residues. In the end, we obtained 184,223 sequences from PDB v2 and 563,129 sequences from UniProt.

### 2.2 Data collection

Antimicrobial peptide data sets were constructed for our model. They key parameters of our model were restricted to activity, assay, function, mechanism target, target organisms, tissue specificity, virus, and amino acid distribution (Table S1 in Supporting information and Fig. 1). The target organisms’ classes were fungus, Gram-positive bacteria, Gram-negative bacteria, insects, mammalian, mollicute, nematodes, parasites, protista, and viruses. The target mechanisms were lipid bilayer, replication, virus entry, DNA/RNA, cytoplasmic proteins, assembly, virucidal, membrane protein, surface glycoprotein, release, and unknown.

Since we have removed known AMPs from the data obtained from the PDB and UniProt databases, we assume that the sequences from the PDB and UniProt databases do not have antimicrobial activity. Table S1 shows the distribution of values across the elements (i.e., target microbes, target mechanisms, MIC_50_, and sequence length). For sequences collected with multiple activity measurements against one or more microorganisms, the measurements with compatible units were uniformly converted to μg/mL. The general antimicrobial activity of the sequences is represented by the arithmetic mean.

As indicated in Fig. 1, the distribution curve of the amino acid with a yellow line exhibited approximately similar trends as the original training sequence. By contrast, the distribution plot of the amino acid generated from the Transformer model displayed a significant difference (Fig. 1). The similarity of the amino acid distributions from the original sequences to the standard normal distribution had a p-value of 0.917, with 0.712 for LSTM and 0.058 for Transformer, indicating that none of these sequences satisfied the normal distribution. Hence, we used the Mann–Whitney U-test and the Kruskal–Wallis H-test, which resulted in LSTM-ori_sequences (original sequence): p-value (0.989, 0.978) and Transformer-ori_sequences: p-value (0.027, 0.026). The significant difference suggests that the LSTM model maintained the stability from the training sequences, and the Transformer model harbored the novelty. To combine the stability of LSTM and the novelty of Transformer, we integrated them and designed novel antimicrobial peptide sequences.

(A) LSTM architecture details. The cell units had a total size of 512 and two layers. The 20 % dropout rate was applied to all Dropout layers. All dense layers are connected to a leaky ReLU (rectified linear unit) activation function behind except for the last layer, which is connected to a sigmoid activation function. Then, the identifying part used the QSAR model, which predicted the function of the generated peptides.
(B) Transformer architecture details. Its central stack consists of six convolutional layers, each with a convolutional kernel of 3, using an exponential dilation rate. Except for the last layer, all the layers use leaked ReLU activation. The last convolutional layer uses a hyperbolic tangent activation function with a convolutional kernel of size 1.
(C) AMPTrans-lstm is a deep generative model and composed of three modules: Transformer, LSTM, and Identifying part. We pre-trained the transformer and LSTM models on a large data set and fine-tuned them on our small data set. During the generation of new samples, we generated some peptide sequences through LSTM sampling and then inputted them into the Transformer model for decoding the novel peptide sequence. Afterward, the trained QSAR model was employed to predict the function of the peptide sequences.

### 2.3 AMPTrans-lstm model

**Fig. 2:**
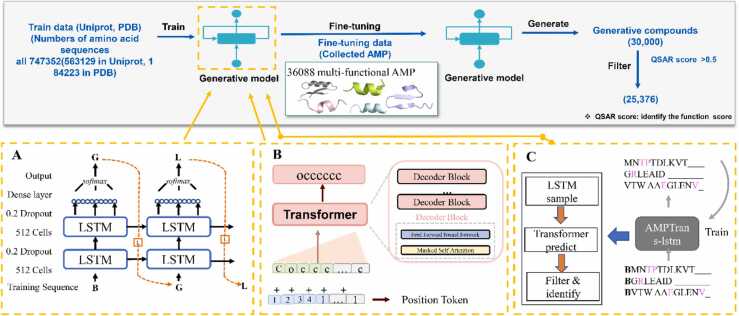
(A) LSTM model, (B) Transformer model, and (C) AMPTrans-lstm architecture.

AMPTrans-lstm was constructed with three neural networks: Transformer, LSTM, and Identifying part (Fig. 2C). During the generation step of peptide sequences, AMPTrans-lstm was fused with two different learning models, which means the output of the LSTM model was used as the input of the Transformer model to generate a new sequence. The Identifying part included two classifiers: (1) QSAR random forest (RF)[54] and (2) QSAR support vector machine (SVM) [55]. Both classifiers were based on pharmacophoric descriptors. The result was the mean obtained from the two prediction scores.

We trained Transformer and LSTM for 100 epochs, where they showed all 747,352 (563,129 in Uniprot and 184,223 in PDB) amino acid sequences in each epoch. Then, we performed fine-tuning on our collected data, where all 36,088 AMP sequences were adjusted in each epoch.

Training was preceded with a batch size of 64 samples irrespective of their origin (either from the AMP set or non-AMP set). The training signal for Transformer and LSTM was provided by CrossEntropyLoss, and the Matthews correlation coefficient (MCC) was used to evaluate the QSAR model. The Transformer model was regularized using Dropout and LayerNorm. LSTM was regularized by L2 regularization and Dropout, which is proven to enhance the stability and generalization of the training. In the current configuration we used (NVIDIA GeForce RTX 3090 24 GB and NVIDIA A40 48 GB), each epoch lasted for roughly 24 s, adding up to 12 GPU hours for 100 epochs.

We trained LSTM and Transformer separately then combined them for final generation in the same datasets. This training approach was selected for the following reasons. A single LSTM model is suitable for small and medium-sized training sets, but it has obvious deficiencies in capturing the context dependence of long sequences and too similar generated sequences resulting from overfitting. Meanwhile, the Transformer model shows good performance in medium and large-sized training sets and has less limitation in capturing the context dependence of long sequences; however, it is difficult to be trained successfully. We hoped to combine the advantages of the two models and generate novel and valid sequences from known AMPs.

## 3 Results and discussion

### 3.1 Training stability

**Fig. 3:**
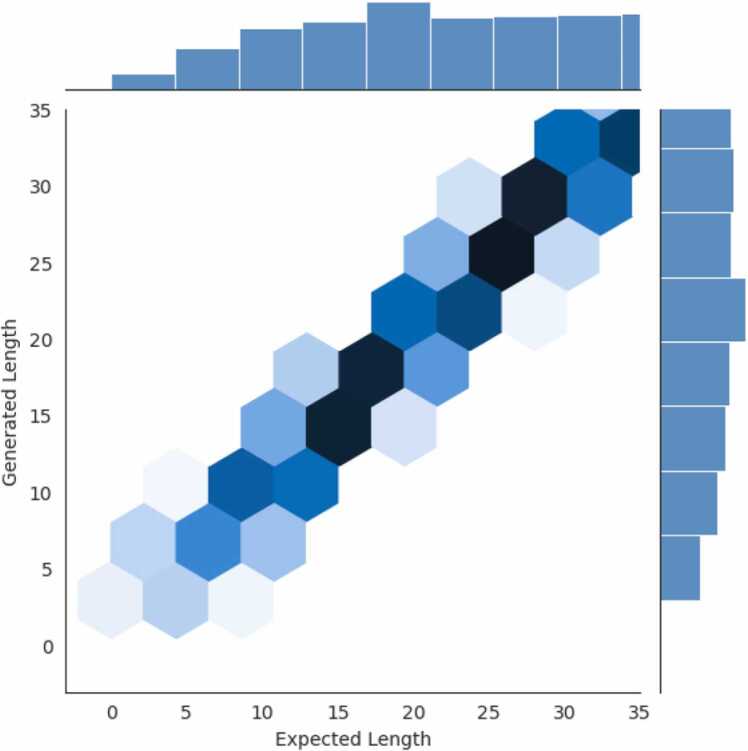
Generated sequence length vs. expected sequence length.

Training the Transformer model is difficult because it requires a massive corpus. Therefore, we employed a heuristic criterion with two different conditions to determine whether specific trial is successful. First, the Transformer model need to generate sequences with character-level entropy that falls between 2 and 4. This condition eliminates models that tend to generate unrealistic sequences with too low or too high FASTA character diversity. For reference, the mean of character-level entropy [56] across our training AMPs, non-AMPs, and their combination was about 2.5, 3.54, and 3.4, respectively. This condition resulted in low character-level entropy (usually close to zero), and the models were clearly useless for generating true AMP candidates. Second, in the LSTM model, our training success criterion needs a successful generator to consider both the sequence length provided in the input data and the correct amino acid sequences. Qualitatively, this means that the lengths of almost all the generated sequences should be similar to the dictated sequence lengths (within± 3), which is visualized in Fig. 3.

The observed ∼ 50 % training success rate increased the number of resources that required to train new iterations of Transformer, a relatively stable model. Based on the estimation provided in the AMPTrans-lstm model section, it takes an average of 150 GPU hours (nearly 7 day) to obtain a reliable model. However, the time needed to train a single model can be reduce to merely 15 h through naively parallelization.

Although it is inconvenient, the low training stability is not a terrible issue because an arbitrary number of AMP candidates can be generated through an obtained quality model. Moreover, the training duration can be shortened from original 100 epochs to 50 epochs because all quality models had passed the criteria at that point. Compared with the original Transformer model, ours can be trained well. This difference indicates that the qualities of the training dataset, rather than the elements of the Transformer architecture, may be the primary reason. We hypothesize that the poor quantity of input data and few samples in our training set (relative to the human language) maybe have contributed to the instability of model training.

**Fig. 4:**
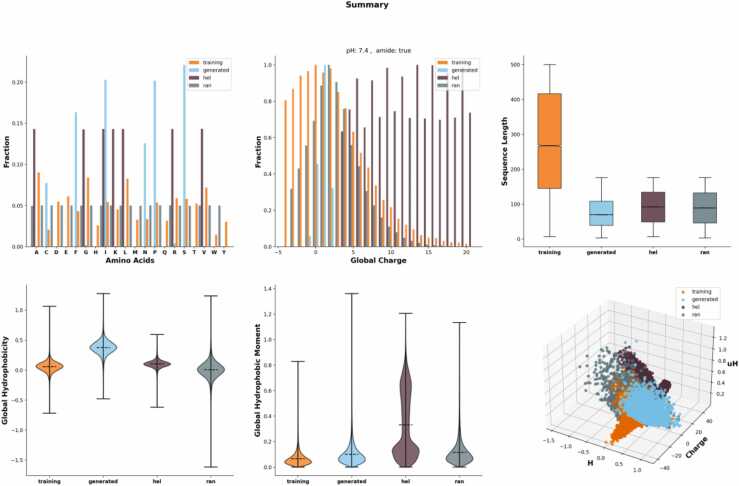
Distributions of generated amino acids by the LSTM model vs. non-generated AMP sequences.

As indicated in Fig. 4, the distribution of trained sequences was created by including 72,000 sequences arbitrarily selected from the training set. The distribution was as follows: 50 % was taken from AMP sequences and other 50 % from non-AMP (other peptide or protein) sequences. The model that used for generating these sequences was stochastically selected from the set of successfully trained LSTM models. The distribution of sequences obtained from the random set (ran) and amphipathic helix set (hel) was created from a sample of 36,000 sequences that were arbitrarily drawn from the random comparison set and the amphipathic helix comparison set. In short, the distribution of generated sequences by LSTM was highly similar with that of our trained set.

### 3.2 Physiochemical similarity

To be applicable to the discovery and design of AMPs, it’s needed to validate the quality of the generator model and the physiochemical properties of the generated candidates. However, experimentally validating the ability of the generator to create sequences that follow the target microbe, MIC_50_ (50 % minimum inhibitory concentration) values [57], and target mechanism is considerably expensive. Alternatively, we focused on comparisons between the easily measurable physiochemical properties of the approved and generated amino acid sequences.

We observed considerable similarity between the amino acid distributions of the training and LSTM-generated AMP sequences, which barely differed by not more than 2 % for most of all the 20 natural amino acids (Fig. 1). The most significant discrepancies were from leucine (L), lysine (K), and serine (S), which were highly prevalent in the trained sequences by 1.6 %, 1.2 %, and 0.8 %, respectively. By contrast, two amino acids, glycine (G) and methionine (M), were 1.0 % and 0.99 % prevalent in the generated AMP sequences, respectively. These small differences suggest a consistent novelty between the known AMPs and generated peptides. However, the large difference between the real AMP and the generated sequences by the Transformer model created massive novel sequences. Here, by default, the sequences displayed were generated by LSTM.

As indicated in Fig. 1, the appearance frequency of single amino acids was investigated, but we noted a complex grammatical structure of peptide feature. We investigated this higher-order organization through generalized word shifts, a method extended the simple analysis performed at the character level to sub-sequences of random length. This method measures the contribution of different sub-sequences to a divergence measure between two groups of sequences and then highlight the largest contributors.

**Fig. 5:**
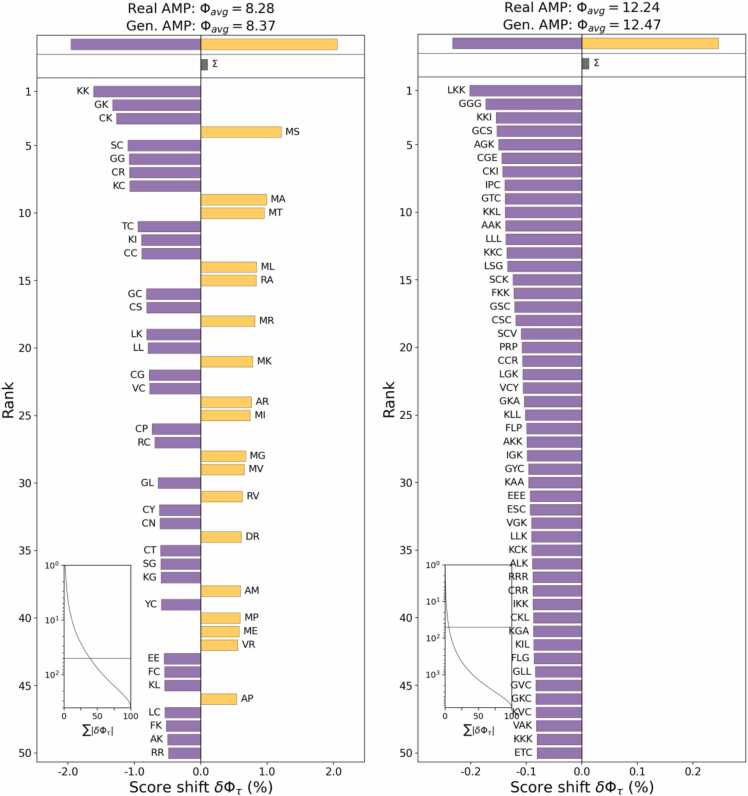
Shannon’s entropy [59] divergence between the distributions of length 3 (right) and length 2 (left) sub-sequences of FASTA characters in AMPs created by the LSTM generator (generated) or AMPs from the training set (real). The purple bars indicate the high prevalence of a particular sub-sequence in the real AMPs while the gold bars indicate the high prevalence of a particular sub-sequence in the generated AMPs. In the top of each panel, the two values indicate the average entropy of each group.

In Fig. 5, we provide word shifts between the real AMPs and generated AMPs for sub-sequences of lengths 3 and 2. Those sub-sequences that common in the generated peptides mostly involved one or more amino acid instances of the A, G, or L. Similarly, those sub-sequences that common in the real peptides tended to involve the L or A amino acid. The two observations reinforce the analysis results at the character level. Most of the sub-sequences in both plots featured a positive charge and hydrophobicity, which coincide well with the known properties of α-helical AMPs. In the sub-sequence shift with a length of 2, the GL and MI motifs were particular interesting because they were often part of those hinge-like structures that near the bends or kinks in proteins.

For reference, the sub-sequences distribution was obtained from a uniformly random sequence set with a maximum entropy of ∼ 12.57 for length 3 sub-sequences and ∼8.38 for length 2 sub-sequences. These two groups (Real AMP and Gen AMP) in both plots featured lower entropy than the maximum (8.38 and 12.57). Thus, meaningful structures were expected in each group. In the lower-left corner of each panel, the cumulative distribution function (CDF) [58] plot indicates that the top 50 contributors to the divergence only accounted for ∼ 5 % (right) and ∼ 37 % (left) of the total divergence; thus, these two distributions were extremely flat.

### 3.3 Sequence diversity

**Fig. 6:**
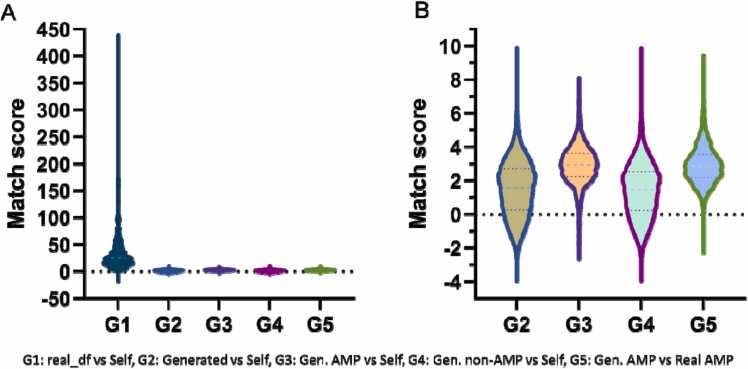
Letter-value figures showing the distributions of match scores that obtained from comparisons between distinct groups of sequences (G1: real_df vs. Self, G2: Generated vs. Self, G3: Gen. AMP vs. Self, G4: Gen. non-AMP vs. Self, G5: Gen. AMP vs. Real AMP). The central horizontal line in each column represents the median value. Each box extending from the median line indicates a percentile that is a half-step between the starting percentile and the terminal percentile in that direction. The dots in the tails represent outliers, in this case, approximately − 12.7 to 0 of the most extreme values in each tail.

When proposing candidate AMPs, the generated candidates must have high diversity and substantial novelty relative to those known AMPs. Those generators that produce sequences with low diversity, can run into the same sampling problems as the extended predictive models discussed earlier. A generative model that cannot produce novel sequences relative to known sequences will be less useful for discovering new AMPs. Here, we quantified the relative similarity of two sets of sequences through the global alignment algorithm [60]. Obtained between a pair of bags, the distribution of alignment scores indicates the relative similarity of the bags, and more similar bags receive higher scores. Fig. 5 shows letter-value plots that summarize the scores obtained by comparing the generated sequences, generated AMPs, generated non-AMPs, and training AMPs to themselves. Moreover, the final letter-value plot shows the distribution of global scores obtained by comparing the training and generated AMP sequences. In Fig. 6, the first distribution shows the match scores obtained by comparing the dataset of training AMPs with itself. The median value of the distribution of match scores for training AMPs was approximately 25 times that of the distribution for the generated AMPs, which indicates that the set of generated AMPs was more diverse than the dataset of training AMPs. When we compared the training AMPs directly with the generated AMPs (the final distribution), we found the lowest median match score observed thus far. This median match score indicates that the generated AMPs were more novel when comparing with the training AMPs. The training AMP score distribution features had upper percentile scores and higher median than others distribution under consideration, which indicates that the training AMP set had relatively lower sequence diversity. The median score of 25 and mean score of 35.03 indicate low sequences diversity, especially compared with the generated AMP sequences that featured a median score of 2.93 and mean score of 2.92. The generated non-AMP sequences had a lower level of diversity relatively to the AMP sequences that had a median score of 1.44 and mean score of 1.3. The combined set of generated sequences obtained slightly higher scores than the non-AMPs separately, with a median of 1.57 and mean of 1.5, which maybe indicate a slight chemical overlap between these two groups or simply by chance. Compared with the training AMPs, the generated AMPs had the lowest scores observed, with a median of 6.4 and mean of 6.5, indicating that these generated AMPs were relative novel.

### 3.4 Estimated antimicrobial activity

We used the predictive models (QSAR models) to estimate the probability that the generated sequences would have antimicrobial activity. We generated 36,000 AMP candidates from AMPTrans-lstm and evaluated them with each of the two predictive machine learning models (SVM and RF). The scores of each sequence obtained from these predictions are given in Supporting information (QSAR model prediction scores). The results of the evaluation of the QSAR model are summarized in Table 1, Table 2, which show that the QSAR model successfully predicted the experimentally validated AMPs.

**Table 1:** Prediction of the expected antimicrobial properties of samples generated by AMPTrans-lstm using machine learning models based on random forest.

	CV0	CV1	CV2	CV3	CV4	CV5	Mean
**MCC**	0.92	0.91	0.92	0.91	0.91	0.92	0.91
**Accuracy**	0.96	0.96	0.96	0.96	0.95	0.96	0.96
**F1**	0.96	0.96	0.96	0.96	0.96	0.96	0.96
**Roc_auc**	0.96	0.96	0.96	0.96	0.95	0.96	0.96
**FDR**	0.05	0.05	0.05	0.05	0.05	0.05	0.05
**Sensitivity**	0.97	0.96	0.97	0.96	0.97	0.96	0.96
**specificity**	0.95	0.95	0.95	0.95	0.94	0.95	0.95

Note: CV0–CV5 refer to the fold of cross validation, the average error is less than 1 % by repeating three times.

**Table 2:** Prediction of the expected antimicrobial properties of samples generated by AMPTrans-lstm using machine learning models based on SVM.

	CV0	CV1	CV2	CV3	CV4	Mean
**MCC**	0.9	0.89	0.9	0.89	0.9	0.9
**Accuracy**	0.95	0.95	0.95	0.95	0.95	0.95
**F1**	0.95	0.95	0.95	0.95	0.95	0.95
**Roc_auc**	0.95	0.95	0.95	0.95	0.95	0.95
**FDR**	0.07	0.07	0.07	0.07	0.06	0.07
**Sensitivity**	0.97	0.96	0.97	0.97	0.97	0.97
**Specificity**	0.93	0.93	0.93	0.93	0.93	0.93

Note: CV0–CV4 refer to the fold of cross validation, the average error is less than 1 % by repeating three times.

## 4 Conclusions

In this study, we introduced AMPTrans-lstm, which combines LSTM and Transformer models and allows for the customized generation of peptides with various degrees of antimicrobial properties. We demonstrated that AMPTrans-lstm can be trained successfully by employing the collected dataset of AMP (our AMP data sets) and non-AMP (public database) data. The results of our analysis involving an extensive comparison between generated peptides and known AMPs indicated that the sequences generated by AMPTrans-lstm were more diverse and innovative than the training data but still preserved the crucial AMP features. We estimated the real success rate of AMPTrans-lstm to be between 30 % and 50 %. If that is the case, then AMPTrans-lstm presents a considerable improvement of over 10 % in terms of success rate for designing an active AMPs compared with traditional design methods. The sequences generated by AMPTrans-lstm were much more likely to be labeled as having antimicrobial properties than the sequences generated at random when evaluated using a suite of predictive machine learning models.

Our model has many valuable features, but it also has some limitations that need to be addressed in future studies. First, it’s needed to improve the lower training stability of the Transformer module to reduce training costs. Second, additional validation is needed to ensure that AMPTrans-lstm is responsive to manipulations of the target microbe and target mechanism prediction. Lastly, to evaluating the quality of the generative models, others quantitative methods should be added to model development and performance comparisons.

With the fast evaluation methods available at present, we have plan for experimentally validating the antimicrobial activity of several promising peptides designed by AMPTrans-lstm. AMPTrans-lstm, which is based on Transformer and LSTM models, contributes to an area where generative models are highly prevalent. In addition, we plan to open the source code of AMPTrans-lstm, thus allowing the general community to modify and use it for the design and discovery of novel and effective AMPs.

## CRediT authorship contribution statement

**Jiashun Mao, Shenghui Guan, Yongqing Chen:** Conceptualization, Methodology, Software, Data curation, Writing – original draft. **Amir Zeb, Qingxiang Sun, Ranlan Lu:** Visualization, Investigation. **Dongsheng Cao:** Supervision. **Jie Dong:** Software, Validation. **Jie Dong, Jianmin Wang, Dongsheng Cao:** Writing – review & editing.
